# Supplementary material for: Accumulation of 4-Hydroxynonenal Characterizes Diabetic Fat and Modulates Adipogenic Differentiation of Adipose Precursor Cells
Source: Int J Mol Sci. 2023 Nov 23;24(23):16645. doi: 10.3390/ijms242316645 (PMC10705911; doi:10.3390/ijms242316645)
Supplement: Supplementary file 1 [file ijms-24-16645-s001.zip › ijms-2669950-supplementary.pdf]

Supplementary Figures

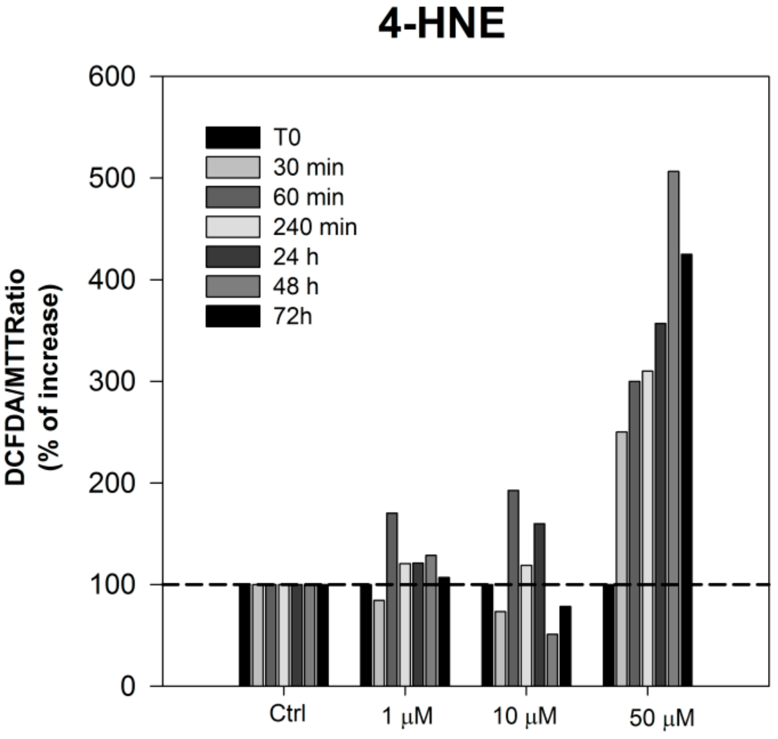

Figure S1. Intracellular ROS levels in human ASCs after challenging with increasing concentrations of 4-HNE. Data are adjusted for cell viability as determined by MTT test and expressed as percentage of optical density relative to control test (Ctrl) that was considered as 100%.
